# A comparative study on anterior teeth retraction-related hard and soft tissue changes with physiologic anchorage control technique

**DOI:** 10.1186/s40001-024-01670-5

**Published:** 2024-02-09

**Authors:** Jianqiao Yuan, Zimeng Zhuang, Longlong Niu, Yuelan Zhang, Shuxia Cui, Hong Su, Gui Chen, Xin Zhang, Bing Han, Si Chen

**Affiliations:** 1 Department of Orthodontics, Peking University School and Hospital of Stomatology & National Center of Stomatology & National Clinical Research Center for Oral Diseases & National Engineering Research Center for Oral Biomaterials and Digital Medical Devices, Beijing, 100081 China; 2 Department of Orthodontics, The First Affiliated Hospital of Zhengzhou University, Zhengzhou, 450052 China; 3 Department of Stomatology, Handan Third People’s Hospital, Handan, 056001 China; 4 First Clinical Division, Peking University School and Hospital of Stomatology, Beijing, 100034 China

**Keywords:** Physiologic anchorage control, Anterior teeth retraction, Soft tissue profile, Protrusive profile, Temporary anchorage devices

## Abstract

Aim of this comparative cross-sectional study was to evaluate the effect of anterior teeth retraction and related hard and soft tissue change under physiologic anchorage control in patients with chief complain of protrusive teeth. 68 Class I or II orthodontic patients undergoing four-premolar extraction and requiring maximum or medium anchorage were included. Patients were treated with physiologic anchorage control technique (PASS group, *n* = 34, 18.6 ± 7.7 years, 10 male and 24 female) and self-ligation technique (Damon group, *n* = 34, 17.5 ± 5.4 years, 13 male and 21 female), respectively. TADs were used for anchorage reinforcement in Damon group. Pre- and post-treatment cephalograms were collected. Twenty-six skeletal, dental and soft tissue items were measured and analyzed using a blinded method. *T* test and paired rank-sum test were used for statistical analysis. The baseline characteristics were similar between groups (*P* > 0.05). After treatment, inter-group comparison showed statistically significant differences in the decrease of skeletal measurements ∠ANB (− 0.73 ± 1.05° in PASS group and − 0.25 ± 0.84° in the Damon group), Wits value (− 2.56 ± 2.29 mm in PASS group and − 0.47 ± 2.15 mm in Damon group) and soft tissue measurement UL-E (− 2.75 ± 1.36 mm in PASS group and − 2.03 ± 1.30 mm in Damon group) and the increase of FCA and Z angle, which was 2.03 ± 2.12°and 9.52 ± 4.78°in PASS group and 0.97 ± 2.12°and 6.96 ± 4.43°in Damon group, respectively (*P* < 0.05). Our results indicated that significant anterior teeth retraction and profile improvement could be achieved with PASS technique without additional anchorage devices. Appropriate application of physiologic anchorage control could reduce the dependence of TADs for anterior teeth retraction.

## Introduction

Protrusion is a common chief complaint in Chinese Orthodontic patients. Extraction of premolars is usually adopted in cases with the chief complaint of protrusion. The prevailing protrusive profile results in a higher extraction rate in orthodontic treatment for Chinese than in other populations [1, 2]. To better retract the anterior teeth to reduce the protrusion, anchorage is required to be reinforced in clinic.

Strong anchorage requires to minimize the forward movement of the posterior teeth, so as to provide more space for retraction of the anterior teeth. However, molar anchorage loss could occur during the early stages of alignment with preadjusted appliance [3, 4]. The classical Tweed edgewise technique utilizes the J-hook headgear and tip-back bend in stainless steel wire to assist molar anchorage preparation before anterior teeth retraction [5]. Whereas in straight wire technique, the most commonly used archwire for initial alignment is Niti wire, which has a shape-memory alloy characteristic [6] and cannot accommodate the tip-back bends needed for molar anchorage preservation. Commonly used intraoral and extraoral anchorage reinforcement devices include Nance arch, transverse palatal rod, facebow, temporary anchorage devices (TADs) and other mechanical devices [4]. Among all these devices, TADs as an absolute anchorage in the bone are more reliable and widely used in clinic because of its small size and less dependence on patient compliance [7]. However, TADs may be rejected by some patients and can only be placed in sites with optimal bone condition [3]. Besides the possible failure and repeated insertion of TADs, the improper utilization of TADs for excessive teeth retraction can impair the health of patients [7].

**Fig. 1:**
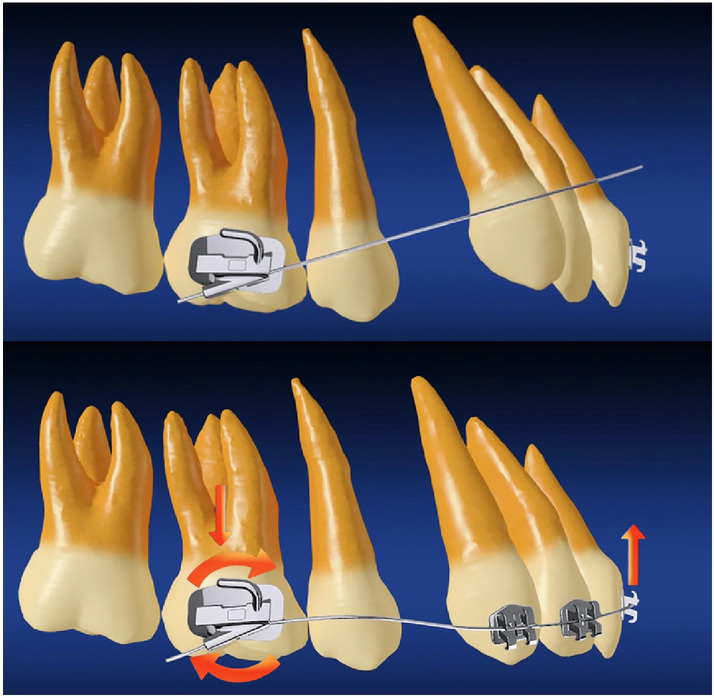
Crossed buccal tube (XBT). (top) with insertion of the archwire into the -25-degree auxiliary tube, anterior archwire will locate gingivally to the anterior teeth; (bottom) after anterior archwire engaging in the brackets, a tip-back moment will be generated on molar to reinforce molar anchorage

**Fig. 2:**
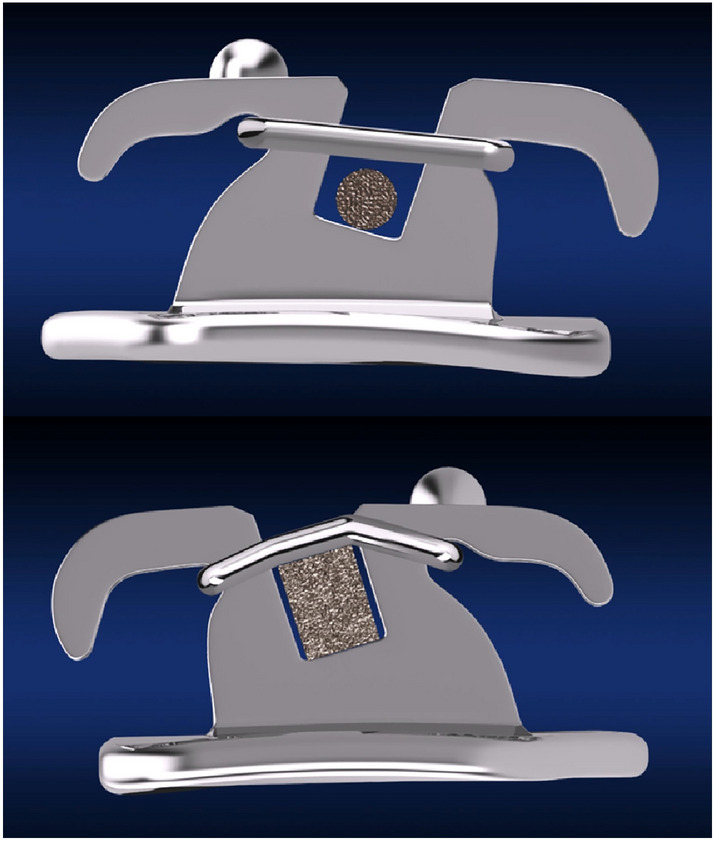
Multilevel low-friction (MLF) bracket. (top) low friction status (round archwire); (bottom) full expression of the prescription status (rectangular archwire)

The Physiological Anchorage Spee-wire System (PASS) was designed to optimize the natural anchorage preservation for better anchorage control. Different from previous mechanical anchorage reinforcement methods, PASS technique enhances anchorage and facilitate teeth movement based on physiological factors [7, 8]. The maxillary first molar moves forward about 2 mm without orthodontic treatment [9], which is naturally anchorage loss. The crossed buccal tube (XBT) maintains the tip-back position of the first molar for anchorage reinforcement from the initial alignment (Fig. 1). Therefore, the naturally anchorage loss could be prevented. During the process of space closure, keeping the tip-back position of molars will be useful to prevent molar anchorage loss, which is similar to the anchorage preparation in Tweed-Merrifield philosophy [5]. Multilevel low-friction (MLF) bracket allows both less friction during alignment and better torque control during anterior teeth retraction (Fig. 2).

In this study, cephalometric analysis was used to investigate whether PASS method can effectively retract anterior teeth and reduce profile protrusion without auxiliary anchorage devices compared with the self-ligation method. Damon system was selected as the control group. In Damon group, TADs were used for anchorage reinforcement in half of the cases. The null hypotheses tested were that there is no difference between the PASS and Damon methods.

## Materials and methods

This study was a comparative cross-sectional cephalometric study and approved by the Biomedical Ethics Committee of Peking University School and Hospital of Stomatology (PKUSSIRB-2013050). The sample size was calculated by the statistical software (G*power, Germany). Two-tailed paired *t* test was adopted. The significant level was α = 0.05. Cohen's d was 0.5. The power of test was 0.8. The ratio of sample sizes in these two groups was 1:1. Statistical measurements showed that the sample size of 34 patients in each group was sufficient for statistical needs.

### Inclusion and exclusion criteria

The inclusion criteria were patients ① in permanent dentition (11 – 41 years); ② with a Class I or II molar relationship; ③ who had four bimaxillary first premolars or two upper first premolars and two lower second premolars extracted, and with medium- or maximum-anchorage that was identified according to the previous study [3]; ④ with pre- and post-treatment cephalograms taken by the same X-ray machine.

The exclusion criteria were patients ① had previous orthodontic treatment; ② with severe periodontitis; ③ who took orthognathic surgery; ④ had molar distalization for extra space; ⑤ had missing or impacted permanent teeth (except third molars); ⑥ had systematic diseases.

### Selection and grouping of the sample

Thirty-four patients (18.6 ± 7.7 years, range from 11 to 41 years; 10 male and 24 female; 19 ≤ 18 years and 15 > 18 years; 21 Class I and 13 Class II) who had undergone premolar extraction treatment with PASS technique (PASS™ appliance, Shinya, Hangzhou, China) in the Department of Orthodontics, Peking University Hospital of Stomatology from January 2014 to January 2020 were selected as PASS group. Thirty-four patients (17.5 ± 5.4 years, range from 11 to 34 years; 13 male and 21 female; 20 ≤ 18 years and 14 > 18 years; 23 Class I and 11 Class II) who had undergone premolar extraction treatment using self-ligation technique (Damon^™^ Q, Ormco, USA) at the same period in the Department of Orthodontics, The First Affiliated Hospital of Zhengzhou University, were selected as Damon group.

Treatment protocols were defined according to the general application of Damon Q self-ligation appliance and PASS appliances. Initial levelling and alignment were performed with round copper nickel titanium archwires. Space closure was performed using rectangular 0.019 × 0.025-in stainless steel wire as working wire and TADs for anchorage reinforcement in Damon group. TADs for anterior tooth retraction in Damon groups were inserted in the buccal space between the second premolars and the first molars. Powerchain providing a traction force of ~ 150 *g* per side was applied for space closure and attached to the molars or TADs. Besides, there were four cases in Damon group using TADs in the anterior maxilla for vertical control. In PASS group, 0.018-in round stainless steel wire with helical loop and molar mesial tip-back bend was used for initial space closure and then 0.018 × 0.025-in stainless steel wire with curve of Spee was used for remaining space closure. Cephalograms were taken before treatment (T0) and immediately after treatment (T1). All the records were anonymized and de-identified prior to analysis.

**Fig. 3:**
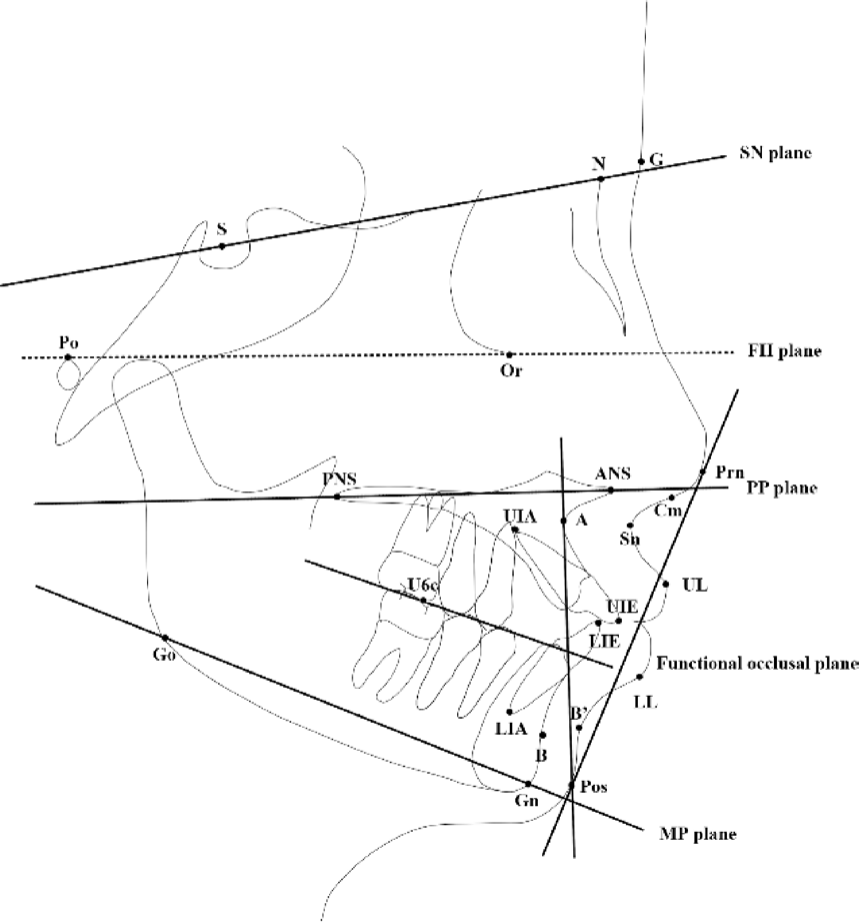
Landmarks. *S* sella, *N* nasion, *Po* porion, *Or* orbitale, *Prn* pronasale, *Sn* subnasale, *G* glbella, *UL* upper lip, *LL* lower lip, *B*’ point B in soft tissue, *Pos* Pogonion in soft tissue, *Gn* gnathion, *Go* gonion, *ANS* anterior nasal spine, *PNS* posterior nasal spine, *A* point A, *UIA* the root apex of the upper central incisor, *UIE* the incisal edge of the upper central incisor, *LIE* the incisal edge of the lower central incisor, *LIA* the root apex of the lower central incisor, *B* point B

Pre- and post-treatment lateral cephalograms of the two groups were measured with the same cephalometric software (Dolphin Imaging Version 11.95 Premium, Dolphin Imaging and Management Solutions, USA). Cephalometric analysis for soft and hard tissues were adopted [10, 11]. Landmarks for cephalometric were presented (Fig. 3). All measurements were performed independently by two trained orthodontists using the blind method, the results were averaged. The group assignment was hidden from both of these two orthodontists conducting measurements. A total of 30 lateral cephalograms were randomly selected for inter-rater consistency. Measurement was repeated after 1 month, and Kappa value (≥ 0.75) indicated good consistency. The specific items are listed in Table 2. The sample size was calculated by the statistical software (G*power, Germany). Two-tailed paired *t* test was adopted. The significant level was α = 0.05. Cohen’s d was 0.5. The power of test was 0.8. The ratio of sample sizes in these two groups was 1:1. Statistical measurements showed that the sample size of 34 patients in each group was sufficient for statistical needs.

### Statistical analysis

Results were analyzed by means of SPSS Statistics 26 (IBM, USA). Shapiro–Wilk test (S–W test) was first used to analyze pre- and post-treatment data in each group. Paired *t* test was applied to results in the normal distribution and paired rank-sum test was for the abnormal distribution. Standard deviation of results between two groups was analyzed by Levene test. Two independent-sample *t* test was used when standard deviations were equal and Welch *t* test was applied when standard deviations were not same. The significant level was α = 0.05 (two-tailed) and *P* value less than 0.05 was considered significant.

## Results

The gender ratios of PASS group and Damon group were comparable, and no statistically significant differences were observed (Table 1). Comparisons of cephalometric results in PASS group and Damon group before and after treatment were listed in Table 2. Before the orthodontic treatment, all items between the two groups did not have statistically significant difference (*P* > 0.05). Among them, ∠ANB was 5.24 ± 2.00° in PASS group and 5.07 ± 1.90° in Damon group. Overjet was 5.01 ± 2.66 mm in PASS group and 5.24 ± 2.16 mm in Damon group. The distance between the upper lip and E line (UL-E) was 2.40 ± 1.84 mm in PASS group and 2.04 ± 1.68 mm in Damon group. The angle of MP–SN was 37.13 ± 5.06° in PASS group and 38.24 ± 6.68° in Damon group. The angle of MP–FH was 29.13 ± 5.12° in PASS group and 29.69 ± 6.17° in Damon group. These results indicated that the lateral profiles and the vertical skeletal patterns were similar between the two groups, which represented patients of the two groups had good comparability before the treatment.

**Table 1:** Gender distribution in PASS group and Damon group

Group		Pass group (*n* = 34)	Damon group (*n* = 34)	χ^2^	*P*
Gender	Male	10	13	0.591	0.442
	Female	24	21

**Table 2:** Comparisons of cephalometric results in PASS group and Damon group pre- and post-treatment

Variable	PASS group (*n* = 34)					Damon group (*n* = 34)					*P* value between two groups	
	Pre-Tx	Post-Tx	change	*t* value	*P* value	Pre-Tx	Post-Tx	Change	*t* value	*P* value	Pre-Tx	Post-Tx
***Skeletal***												
∠SNA (°)	82.73 ± 2.65	82.07 ± 3.02	− 0.65 ± 0.97	3.951	0.000**	81.71 ± 3.98	81.61 ± 4.00	− 0.10 ± 1.04	0.577	0.568	0.220	0.027*
∠SNB (°)	77.49 ± 3.08	77.57 ± 3.24	0.08 ± 1.08	− 0.429	0.670	76.64 ± 4.12	76.78 ± 4.48	0.14 ± 1.02	− 0.816	0.420	0.338	0.804
∠ANB (°)	5.24 ± 2.00	4.51 ± 1.93	− 0.73 ± 1.05	4.050	0.000**	5.07 ± 1.90	4.82 ± 1.90	− 0.25 ± 0.84	1.7431	0.091	0.729	0.041*
Wits value (mm)	1.41 ± 3.46	− 1.17 ± 2.15	− 2.56 ± 2.29	6.615	0.000**	1.82 ± 2.70	1.34 ± 2.42	− 0.47 ± 2.15	1.286	0.207	0.596	0.000**
MP–SN (°)	37.13 ± 5.06	36.74 ± 5.48	− 0.39 ± 1.49	1.518	0.139	38.24 ± 6.68	37.93 ± 7.23	− 0.31 ± 1.55	1.1586	0.255	0.444	0.833
MP–FH (°)	29.13 ± 5.12	28.85 ± 5.27	− 0.28 ± 1.45	1.109	0.275	29.69 ± 6.17	29.79 ± 6.63	0.10 ± 1.52	-0.395	0.696	0.686	0.297
***Dental***												
∠U1–NA (°)	27.90 ± 9.26	17.92 ± 6.66	− 9.98 ± 8.60	6.768	0.000**	29.09 ± 7.10	20.04 ± 8.70	− 9.05 ± 7.56	6.983	0.000**	0.554	0.637
∠U1–PP (°)	120.10 ± 10.07	109.85 ± 6.14	− 10.25 ± 8.51	7.023	0.000**	120.27 ± 6.55	110.48 ± 8.38	− 9.79 ± 7.89	7.235	0.000**	0.936	0.816
∠U1–SN (°)	110.64 ± 9.77	99.98 ± 6.29	− 10.66 ± 8.47	7.341	0.000**	110.78 ± 6.91	101.41 ± 9.21	− 9.37 ± 7.36	7.420	0.000**	0.944	0.506
U1–NA (mm)	6.11 ± 2.31	1.77 ± 1.79	− 4.34 ± 2.06	12.302	0.000**	6.73 ± 2.24	2.42 ± 2.26	− 4.31 ± 2.19	11.448	0.000**	0.262	0.950
U1–AP (mm)	10.85 ± 2.68	4.98 ± 1.99	− 5.87 ± 2.08	10.254	0.000**	11.38 ± 2.40	6.60 ± 2.27	− 4.79 ± 2.10	8.437	0.000**	0.3934	0.003**
U1–PP (mm)	28.38 ± 3.00	28.98 ± 2.98	0.85 ± 2.15	− 2.039	0.027*	28.74 ± 2.56	28.42 ± 3.69	− 0.32 ± 2.36	0.790	0.435	0.599	0.036*
∠L1–NB (°)	33.07 ± 5.76	26.28 ± 6.89	− 6.79 ± 7.46	5.300	0.000**	33.18 ± 7.26	27.07 ± 5.28	− 6.11 ± 7.72	4.618	0.000**	0.943	0.716
∠L1–MP (°)	98.44 ± 7.68	91.97 ± 9.41	− 6.48 ± 7.20	5.248	0.000**	98.31 ± 8.05	92.36 ± 6.55	− 5.95 ± 7.54	4.605	0.000**	0.944	0.770
L1–NB (mm)	8.75 ± 2.16	5.05 ± 1.85	− 3.70 ± 1.93	11.186	0.000**	8.86 ± 2.61	6.03 ± 1.95	− 2.83 ± 2.26	7.312	0.000**	0.850	0.093
L1–MP (mm)	41.06 ± 3.26	39.82 ± 3.72	− 1.24 ± 2.25	3.220	0.003**	41.79 ± 3.48	41.12 ± 3.56	− 0.67 ± 2.22	1.748	0.000**	0.377	0.293
∠U1–L1 (°)	113.79 ± 11.47	131.31 ± 9.31	17.52 ± 11.24	− 9.091	0.000**	112.67 ± 9.81	126.86 ± 10.25	14.19 ± 12.08	6.847	0.000**	0.669	0.243
∠U6–PP (°)	96.45 ± 8.53	97.02 ± 9.50	0.57 ± 6.51	0.260	0.795	98.99 ± 7.54	95.35 ± 6.40	− 3.65 ± 7.46	2.146	0.036*	2.628	0.010*
∠L6–MP (°)	96.96 ± 5.58	99.74 ± 7.56	2.78 ± 5.30	1.725	0.089	97.93 ± 7.23	101.28 ± 6.24	3.35 ± 7.00	2.045	0.045*	0.403	0.688
U6c–PP (mm)	22.76 ± 2.18	23.69 ± 2.19	0.93 ± 1.41	1.755	0.084	22.54 ± 2.78	23.68 ± 2.56	1.13 ± 1.68	1.759	0.083	5.074	0.568
U6c–PNS (mm)	20.87 ± 3.28	23.23 ± 2.76	2.36 ± 2.14	3.210	0.002**	19.50 ± 3.59	23.39 ± 3.09	3.89 ± 2.57	4.789	0.000**	2.816	0.006*
overjet (mm)	5.01 ± 2.66	3.06 ± 0.81	− 1.95 ± 2.61	4.121	0.000**	5.24 ± 2.16	3.28 ± 0.95	− 1.97 ± 2.39	4.800	0.000**	0.567	0.843
MIA (°)	11.42 ± 4.52	10.98 ± 6.37	− 0.44 ± 6.74	− 0.379	0.707	10.27 ± 3.77	9.29 ± 5.31	− 0.98 ± 5.57	1.028	0.312	0.258	0.718
OP–FH	9.51 ± 4.02	12.46 ± 4.14	2.95 ± 3.15	− 5.465	0.000**	9.12 ± 3.71	10.22 ± 4.82	1.10 ± 2.93	− 2.192	0.036*	2.504	0.015*
OP–SN	18.19 ± 4.22	20.99 ± 4.67	2.80 ± 2.91	− 5.599	0.000**	18.16 ± 3.99	18.60 ± 5.55	0.44 ± 3.25	− 0.784	0.439	3.156	0.002**
∠FOP–PP (°)	20.97 ± 7.37	14.82 ± 5.40	− 6.16 ± 6.13	3.925	0.000**	19.86 ± 6.31	15.60 ± 5.71	− 4.26 ± 5.68	2.919	0.005**	1.399	0.166
∠AOP–PP (°)	9.70 ± 4.80	14.25 ± 4.68	4.55 ± 3.41	3.958	0.000**	10.44 ± 4.62	13.31 ± 5.14	− 2.87 ± 4.28	2.421	0.018**	1.897	0.062
∠POP–PP (°)	23.12 ± 8.99	15.70 ± 5.24	− 7.42 ± 6.98	4.158	0.000**	21.38 ± 7.67	14.91 ± 5.23	− 6.48 ± 6.35	4.064	0.000**	0.614	0.541
∠POP–AOP (°)	13.41 ± 8.76	1.44 ± 4.49	− 11.98 ± 7.23	7.091	0.000**	10.95 ± 7.70	1.60 ± 3.92	− 9.35 ± 7.87	6.310	0.000**	1.516	0.134
***Soft tissue***												
NLA (°)	97.55 ± 11.25	100.75 ± 10.15	3.20 ± 7.06	− 2.647	0.012*	96.40 ± 11.90	100.58 ± 12.66	4.18 ± 6.67	− 3.66	0.001**	0.684	0.558
LLA (°)	140.70 ± 13.96	137.99 ± 11.97	− 2.71 ± 10.52	1.501	0.143*	134.65 ± 16.54	133.64 ± 11.12	− 1.01 ± 11.07	0.535	0.597	0.108	0.520
UL-E (mm)	2.40 ± 1.84	− 0.34 ± 1.56	− 2.75 ± 1.36	11.808	0.000**	2.04 ± 1.68	0.01 ± 1.77	− 2.03 ± 1.30	9.077	0.000**	0.402	0.030*
LL-E (mm)	4.41 ± 2.20	0.64 ± 1.67	− 3.77 ± 1.77	12.436	0.000**	4.74 ± 2.15	1.79 ± 2.22	− 2.95 ± 1.65	10.422	0.000**	0.544	0.053
FCA (°)	166.40 ± 6.23	168.42 ± 5.14	2.03 ± 2.12	− 5.581	0.000**	165.98 ± 5.45	166.95 ± 4.79	0.97 ± 2.12	− 2.674	0.012*	0.768	0.043*
Z angle (°)	60.93 ± 7.74	70.45 ± 6.32	9.52 ± 4.78	− 11.616	0.000**	59.42 ± 6.77	66.38 ± 7.82	6.96 ± 4.43	− 9.151	0.000**	0.397	0.025*

Wits value (mm): the distance between perpendiculars from subspinale and supramental onto occlusion plane; ∠U1–NA: the angle between long axis of upper central incisor and N–A line; ∠U1–PP: the angle between the long axis of upper incisor and the palatal plane; ∠U1–SN: the posterior–inferior angle between the long axis of upper incisors and the SN plane; U1–NA (mm): the perpendicular distance from U1 tip to N–A line; U1–AP (mm): the perpendicular distance from U1 tip to A-Pos line; U1–PP (mm): the perpendicular distance from U1 tip to the palatal plane; ∠L1–NB: the angle between the long axis of lower central incisor and N–B line; ∠L1–MP: the angle between the long axis of lower central incisor and the mandibular plane; L1–NB (mm): the perpendicular distance from L1 tip to the N–B line; L1–MP (mm): the perpendicular distance from L1 tip to mandibular plane; ∠U1–L1: the angel between long axes of the upper and the lower central incisors; ∠U6–PP: the antero-inferior angle between the long axis of upper first molar and PP plane; ∠L6–MP: the anterosuperior angle between the long axis of lower first molar and MP plane; U6c–PP (mm): the distance between PNS point and the perpendicular projection point from U6 mesiobuccal cusp to PP plane; U6c–PNS (mm): the distance between PNS point and the perpendicular projection point from U6 mesiobuccal cusp to PP plane; MIA: the angle between the long axis of lower central incisor and the long axis of the mandibular symphysis; OP–FH: the angle formed by functional occlusion plane and FH plane; OP–SN: the angle between the functional occlusion plane and the SN plane; ∠FOP–PP: the angle between the full occlusal plane and PP plane; ∠AOP–PP: the angle between the anterior occlusal plane and PP plane; ∠POP–PP: the angle between the posterior occlusal plane and PP plane; ∠POP–AOP: the angle between the anterior and the posterior occlusal plane; *NLA* nasolabial angle, *LLA* mentolabial angle, *UL-E (mm)* the distance from the upper lip point to the E line, *LL-E (mm)* the distance from the lower lip point to the E line, *FCA* facial contour angle, *Z angle* the angle between FH plane and the line of pogonion and lip point

^*^P<0.05; ^**^ P<0.01

After the treatment, satisfied clinical outcomes were achieved in both groups. Anterior overbite and overjet significantly decreased and the prominence of profile was reduced. In both two groups, items including upper incisor labial inclination and protrusion, lower incisor labial inclination and protrusion, vertical height of lower central incisors, inter-incisal angle, overjet, facial contour angle, and the distance from the upper or lower lip point to the E line, were all significantly improved after treatment.

The comparison between PASS group and Damon group showed significant differences in the following measurements. The decrease of ∠SNA was − 0.65 ± 0.97° in PASS group and − 0.10 ± 1.04° in Damon group (*P* < 0.05). The decrease of ∠ANB was − 0.73 ± 1.05° in PASS group and − 0.25 ± 0.84° in Damon group (*P* < 0.05). Wits value decreased by − 2.56 ± 2.29 mm in PASS group and − 0.47 ± 2.15 mm in Damon group (*P* < 0.001). The change of the inclination of occlusion plane (OP–SN) was 2.80 ± 2.91° in PASS group and 0.44 ± 3.25° in Damon group (*P* < 0.05). The decrease of UL-E line was − 2.75 ± 1.36 mm in PASS group and − 2.03 ± 1.30 mm in Damon group (*P* < 0.05). The increase of FCA was 2.03 ± 2.12°in PASS group and 0.97 ± 2.12°in Damon group (*P* < 0.05). The increase of Z angle was 9.52 ± 4.78°in PASS group and 6.96 ± 4.43°in Damon group (*P* < 0.05).

## Discussion

This study chose self-ligation bracket and TADs as the control group to evaluate the effect of PASS technique on anterior teeth retraction and profile improvement in patients with protruded anterior teeth and convex profile.

Anchorage reinforcement is required to address the convex profile when the extraction space is designed to mainly leave for achieving more retraction of anterior teeth [12]. Anchorage preparation is considered to be the most important step in clinical orthodontics [13, 14]. TADs are recognized to be the prominent anchorage reinforcement devices because of more anchorage preservation than conventional devices [15] and controlled tipping of anterior teeth [16]. This study represented significant improvement of profile in Damon group, which could partly contribute to the low-friction movement and assistance of TADs in half of cases. However, results also showed that the significant improvement of profile also occurred in PASS group without TADs. In PASS group, pre-treatment upper anterior teeth were proclined and protruded. Convex profile was characterized by lip protrusion point locating in front of the E-line (the distance of UL-E was 2.40 ± 1.84 mm, the distance of LL-E was 4.41 ± 2.20 mm). Results showed that after treatment, the anterior teeth were significantly retracted and the protrusion of lip reduced to normal (the distance of UL-E was − 0.34 ± 1.56 mm, the distance of LL-E was 0.64 ± 1.67 mm), which indicated that PASS could achieve significant anterior teeth retraction under good anchorage control. Changes of U1–AP were − 5.87 ± 2.08 mm in PASS group and − 4.79 ± 2.10 mm in Damon group. The sagittal linear movement of U1 was significantly greater in PASS group than in the Damon group, which resulted to the significantly greater change of UL-E (mm). Results showed that PASS technique without extra anchorage reinforcement devices could achieve better profile improvement for patients with protruded teeth.

With classical Tweed edgewise technique, molars are tip-back first to achieve the anchorage preparation position before anterior teeth retraction [5]. However, for the conventional straight-wire method, the upper first molar tends to tip forward during alignment, because the prescription of the buccal tube contains mesial inclination which uses the molar position in natural normal dentition for reference [3]. This tip-forward prescription can lead to early anchorage loss, especially for the initially distal-tipping molars [3].

PASS group utilized the physiologic anchorage control system improved based on the perspective of preventing physiological anchorage loss [17]. PASS technique protects and reinforces the anchorage from the three-dimensional direction. The specific innovations of PASS technique are as follows: ①XBT buccal tube permits molars remaining in the dominant torque position [18], maintains the initial distal tipping of upper molars [19, 20], and changes the direction of the molar eruption from forward and downward to downward and backward. Tip-back angle is also applied to second premolars and second molars. Maxillary molars will attain anchorage preparation when in conjunction with Spee-curve arch wire, which could be used as the anchorage preparation in the closure of extraction spaces and at the meantime, provide more spaces for the incisor retraction. ② By maintaining distal tipping of erupting posterior teeth in adolescent, vertical growth of molars is transformed into the space of arch in sagittal direction, thereby increasing the length of the upper arch, resulting in the effect similar to molar distalization [21].

Previous study systematically reviewed anchorage methods and found the evidence of a preference of any anchorage method lacked sufficient evidence [15]. Moreover, regarding adverse effects of TADs containing tooth root injury and mucosa lesion [22], the use of TADs may be limited in adolescents or patients who require non-invasive treatment. The philosophy of healthy orthodontic treatment should be reflected not only in getting good treatment outcome, but also in minimizing trauma to patients during the treatment and ensuring that patients benefit the most from orthodontic treatment. Therefore, a noninvasive and comfortable anchorage reinforcement method is required. Physiologic anchorage control concept emphasizes the role and influence of growth and biological response should be taken into account to facilitate anchorage reinforcement. The changes of U6c–PNS distance after treatment were 2.36 ± 2.14 mm in PASS group and 3.89 ± 2.57 mm in Damon group, which showed molars had less mesial movement in PASS group (*P* = 0.006). The changes of ∠U6–PP (°) post-treatment were 0.57 ± 6.51° in PASS group and − 3.65 ± 7.46° in Damon group (*P* = 0.010), which indicated molars in PASS groups slightly distally tipped but molars in Damon group mesially tipped. The Changes of ∠U6–MP (°) post-treatment were 2.78 ± 5.30° in PASS group and 3.35 ± 7.00° in Damon group, and changes of ∠U6–PP (°) post-treatment were 0.57 ± 6.51° in PASS group and − 3.65 ± 7.46° in Damon group, which showed statistically significant difference (*P* = 0.010) Results indicated even without the assistance of TADs, PASS technique still performed better in anchorage preservation than Damon system in this study.

Inter-group comparison showed that 12 items among the 35 soft and hard tissue items had statistically significant differences. PASS group showed better results in 10 of the 12 items, among which Wits value change was found to have more significant difference between the two groups (− 2.56 ± 2.29 mm in PASS group and − 0.47 ± 2.15 mm in Damon group, *P* < 0.001). The difference might be related to the clockwise rotation of the occlusal plane after treatment resulted from maintaining distal tipping of molars in PASS group that was corroborated by changes of U6c–PNS distance and ∠U6–PP in the two groups. The statistically significant change of OP–SN (2.80 ± 2.91° in PASS group and 0.44 ± 3.25° in Damon group) confirmed the rotation of the occlusal plane in PASS group. For profile improvement, PASS group demonstrated better improvement in lip prominence reduction (UL-E), the facial contour angle increase (FCA) and the Z angle increase. The better profile improvement contributed to more retraction of anterior teeth and less mesial movement of molars, which represented achieving higher anchorage than Damon group. These results might relate to the anchorage protection in PASS group throughout the treatment.

This study has several limitations. First, lateral cephalograms after the initial alignment were lack due to the requirement of minimizing the radiation exposure. Since we contribute the advantage of anterior tooth retraction and anchorage preservation in PASS group to its maintenance of physiologic anchorage during the alignment process, the evidence during the treatment would further enhance the credibility. Second, though patients between the two groups had similar facial type before the treatment, this study lacked strict adherence to the specific age and gender. In addition, this study did not separately compare the clinical efficacy between males and females. Future studies could take dental casts during the treatment and conduct on a single gender to further certify the physiological anchorage preservation from PASS technique.

## Conclusion

For patients with dental protrusion and related convex profile, PASS technique can effectively retract anterior teeth and improve soft tissue profile. Proper application of PASS technique can achieve healthy, aesthetic and stable therapeutic outcomes, of which tissue response is close to physiological changes.

## Data Availability

The data sets used and/or analyzed during the current study are available from the corresponding author on reasonable request.
